# Quality of Life and Related Factors in Specialists on Pediatric Dentistry and the like Graduated from a Public University: A Mixed Methods Approach

**DOI:** 10.3390/ijerph192013107

**Published:** 2022-10-12

**Authors:** Ariana Amariles-Baena, Catalina Sosa-Palacio, Andrés A. Agudelo-Suárez

**Affiliations:** Faculty of Dentistry, University of Antioquia, Medellin 050010, Colombia

**Keywords:** mental health, pediatric dentists, quality of life, research technics

## Abstract

This study aims to analyze the factors associated with the quality of life (QOL) in pediatric dentistry specialists and the like, graduated in a public university between 1972 and 2018. A mixed study (explanatory sequential design) was conducted. Firstly, a cross-sectional survey (*n* = 62, 51% women) was carried out and complemented with three focus groups (FGs) and four semi-structured interviews (SIs). Descriptive statistics, bivariate analyzes and non-parametric correlation were calculated. A multivariate linear regression was carried out to establish the factors associated with QOL. In FGs, the concept of QOL and the factors that influence it was investigated, by following a qualitative content analysis and later the information was triangulated. The median QOL scores surpass 62 points. The multivariate analysis showed that the factors exerted the greatest influence negatively (decreases the QOL score) were: having an independent or service provision contract (*p* < 0.05), low social support (*p* < 0.001), job dissatisfaction (*p* < 0.01), poor mental health (*p* < 0.01), self-perceived poor general health (*p* < 0.01). The information from the FGs and interviews allow to establish three categories of analysis: (1) QOL and health: multiple facets, determinants, and dimensions; (2) encounters and disagreements between the postgraduate curricular training proposal and the labor and social field of the specialist; (3) an itinerant clinical specialization. The QOL of participants is considered good in general terms and is conditioned by subjective factors, the social environment, and the conditions of their professional work.

## 1. Introduction

The concept of quality of life (QOL) underwent an important historical evolution in both conceptual and methodological aspects and has been used daily in different areas of knowledge, such as health, education, economics, and politics [1]. Several definitions have been proposed for this concept which are unified by the distinction between subjective aspects such as physical, psychological, and social well-being, and objective factors such as material well-being, harmonious relations with the physical and social environment, and self-perceived health [2]. The World Health Organization (WHO) defines QOL as an individual’s perception of their position in life in the context of the culture and value systems in which they live and in relation to their goals, expectations, standards, and concerns [3]. However, more recent authors, have proposed the differentiation of the QOL concept from others such as “health status” and “health-related quality of life” (HRQOL) [4]. In general, as a global term, this construct has been studied in different populations and through different methods and measurement instruments [5].

One of the professions that have perhaps been most affected by the QOL is the dental profession. The restructuring of their practice in recent decades has led to the QOL of these professionals, including specialists, decreasing compared to the dentists of the past [6]. Possibly the most determining factor was the entry into force of the neoliberal economic model in the country, which caused a change in the provision of social services, including dentistry, which further transformed it from being a profession predominantly liberal to entering into the logic of the market economy where the individual interests of various stakeholders put the right to health at risk [6].

At an international level, few specific investigations are focused on the QOL of this professional group [7,8,9]. This may be because the scientific literature has focused on specifically studying some characteristics related to working conditions, occupational risk factors related to dental care, and aspects related to physical, mental, and psychosocial health indicators (for example, the burnout syndrome) [10,11,12]. These conditions could be considered an undirected proxy of QOL, because working for extensive hours or under pressure, or with high workloads and/or demand from patients, affects the health situation of both general dentists and specialists [7,8,9].

Specifically in Colombia, some theoretical and investigative approaches have been made to understand the current situation of the profession in social terms, well-being, quality of life at work, and health [6,13,14,15]. However, it is necessary to expand the scope of research agenda to know the experiential realities of different clinical specialists in dentistry. In this sense, a study published by Muñoz et al. in 2020 was conducted for identifying the factors associated with the QOL in a group of orthodontists from a public university using quantitative and qualitative techniques [16].

The clinical specialization of maxillary orthopedics at the University of Antioquia in Medellin (Colombia) has had various transformations in its curricula in the last decades. Inquiries into the archives of the institution show that this postgraduate course, created in the 1960s as pediatric dentistry has presented different modifications in its name (specialty in pediatric dentistry, specialty in comprehensive child dentistry, clinical specialty in comprehensive child dentistry and maxillary orthopedics, clinical specialty in maxillary orthopedics) [17,18]. Therefore, it is necessary to study this phenomenon in depth, through an analysis recognizing that historical, contextual, social, and individual factors are associated with the QOL and health of these professionals. This research will permit establishing action plans and strategies based on the realities experienced by those directly investigated. In this aspect, mixed studies are well accepted in dentistry since they permit a comprehensive approach to health and social phenomena from statistical aspects and consider the opinions and perceptions of the realities faced and lived by individuals [19].

Accordingly, this study aimed to analyze the factors associated with the QOL of the clinical specialists in pediatric dentistry and the like who graduated from a public university between 1972 and 2018.

## 2. Materials and Methods

### 2.1. Design

**Figure 1:**
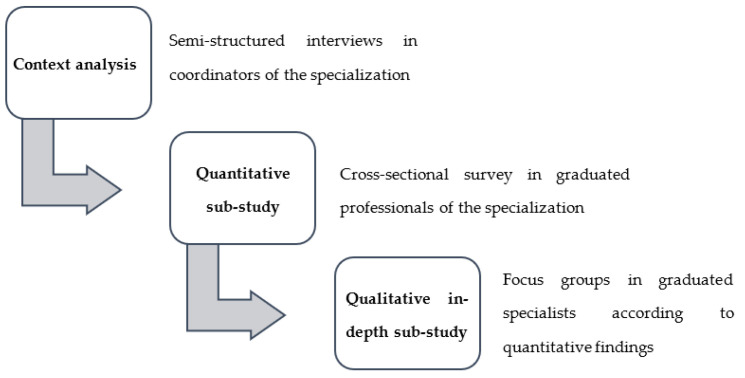
Methodological flow chart for the study design.

Considering that the QOL is a multifactorial and multidimensional element, a mixed study with an explanatory sequential design was proposed [19]. This study consists of the qualitative methods allow explaining the quantitative findings. Nevertheless, at the beginning of the study and as a context integrative complement, the research team decided to conduct four semi-structured interviews (SIs) with the coordinators of the clinic specialization in the study period (Figure 1). This inclusion facilitated the triangulation of the findings, following the proposed methodological route. These interviews were focused on understanding the main characteristics of the educative program and the general perspective about the target population (QOL, labor and social aspects). The study was not preregistered.

### 2.2. Quantitative Sub-Study (Cross-Sectional)

A cross-sectional survey was applied to clinical specialists in pediatric dentistry (according to a diploma named as pediatric dentists, comprehensive child dentistry, and/or maxillary orthopedics specialists) who graduated from the Faculty of Dentistry of the University of Antioquia (Medellín, Colombia). Data of participants were supplied by the institution, and a final sample of 62 participants was obtained (51% females), considering voluntary participation. An online questionnaire was carried out, using the Google Forms platform (available upon request). A pilot study was carried out on a sample of 10 respondents to improve intelligibility, to assess time to completion and internal consistency. The survey was conducted between March and June 2021.

The main outcome was the quality of life (QOL), as measured by the WHOQOL-BREF (acronym for abbreviated World Health Organization quality of life questionnaire) [3]. This is a generic questionnaire to measure QOL created by the study group on quality of life of the WHO and comprises of 26 items distributed on four broad domains: physical (seven items: daily activities, medicinal substances/medical aids, energy, mobility, pain/discomfort, sleep/rest, functional/work capacity), psychological (six items: self-image, negative thoughts, positive attitudes, self-esteem, spirituality/religion/personal beliefs, thinking, learning, memory and concentration), social relationships (three items: personal relationships, social support, and sex life), and environmental health (eight items: financial resources, safety, health and social services, living physical environment, opportunities to acquire new skills and knowledge, recreation, general environment (noise, air pollution, etc.), and transportation). It also contains two QOL and general health items [3,20]. All the items give a raw score, which is transformed to a 0–100 scale, according to the recommendations of the study group. Higher scores indicate higher QOL levels. This questionnaire has been validated and is available in 19 languages, including Spanish [21].

As explanatory variables were included: employment conditions, sociodemographic, sport practice, general health, self-perceived stress, mental health, as measured with the General Health Questionnaire-12 (GHQ-12). This is a self-administered tool that intend to screen current general (non-psychotic) mental health problems and related disorders and commonly used in primary health care. Respondents are asked to indicate the degree to which they have recently experienced different situations related to distress (lack of concentration, capacity of making decisions, unhappiness, among others) [22]. Responses were rated and summed, and individuals with a score of 3 or higher were classified of having poor mental health [23]. The Duke-UNC (University of North Carolina) functional social support questionnaire (Duke-UNC-11) was used to measure social support. This instrument containing 11 items and evaluates perceived functional or qualitative social support received for family, relatives, friends in different situations (visits, help, love/affection, invitations, advice, among others) [24]. Each item is scored on a frequency rating from 1: “Much less than I would like” to 5: “As much as I would like”. The score was calculated by adding up the responses to each item, with a higher score denoting greater social support. The cut-off point for low levels of social support is the 15th percentile, corresponding to a score of 32 [25].

Reliability tests were conducted by the WHOQOL-BREF, GHQ-12, and Duke-UNC-11 instruments. The Cronbach’s alpha coefficient obtained for the three questionnaires were: (1) WHOQOL-BREF: Global: 0.909 and for the domains physical: 0.788; psychological: 0.756; social relationships: 0.731; environment: 0.727; (2) GHQ-12: 0.844; (3) Duke-UNC-11: 0.906 (acceptable in all cases for the study purposes). A descriptive analysis was carried out for all variables. The Kolmogorov-Smirnov test was used for verifying normality distribution in the main outcome. A bivariate analysis was conducted for the scores of the domains of QOL with qualitative explanatory variables and tests of statistical significance were carried out to observe differences among variables according to their nature (Mann-Whitney U test for dichotomous variables, Kruskal-Wallis test for polychotomous variables, and the Spearman correlation for quantitative variables). A linear multivariate regression analysis was carried out in order to evaluate the simultaneous and reciprocal association of the explanatory variables on each of the dimensions of WHOQOL-BREF and to identify possible predictors of their scores. The Stepwise method was used in the case of the four multivariate linear regressions considering the QOL; and according to biological plausibility and previous studies [16], sociodemographic (*n* = 7), work (*n* = 21), and health (*n* = 5) variables were included. For the physical domain, four models were made where the adjusted final only included four predictors with statistical significance (independent contract, provision of services contract, self-perceived health, mental health). For the psychological domain, only one model including a predictor (mental health) was made, eliminating the rest of the variables. In the case of the social relationships dimension, two models were included and the adjusted final included two predictors (self-perceived health, and mental health). Finally, regarding the environment domain, only one model including a predictor (labor satisfaction) was established. Belonging was determined by evaluating the compliance with the assumptions of linearity, non-collinearity and normality, constant variance, and correlation of residuals. All of the analyses used a level of statistical significance of <0.05. SPSS software version 22.0-IBM^®^ was used to carry out all of the analyses.

### 2.3. Qualitative Sub-Study (Focused Ethnographic Perspective)

A qualitative approach was conducted using three focus groups (FGs) and participated fourteen respondents that previously completed the survey (selected for convenience). Fieldwork for interviews and FGs was carried out between October 2020 and March 2022. The research team produced a guide for use in the FGs. The research team produced a guide for use in the FGs that indicated a series of topics to be discussed among participants. The factors that determine the QOL (for or against) were deepened, allowing points of agreement or disagreement in the information, and expanding and clarifying the information that was found in the quantitative survey.

The FGs were conducted by two members of the research team (A.A.-B. and C.S.-P.) and supervised by the third member (A.A.A.-S.), who is specialized in qualitative methods. Considering the pandemic situation because of the COVID-19, remote means were used (Meet-Google and Microsoft Teams). FGs lasted between 60 and 90 min and were digitally recorded and transcribed verbatim. FGs were performed until data saturation was reached, meaning that no new information emerged.

Firstly, in order to identify text fragments and meanings, an initial reading of data by researchers was performed. Narrative content analysis was conducted identifying significant pieces of text and trends of information found in the participants’ discourse. Data analyses were conducted by the research team, who examined and compared their analyses. Transcribed data were imported into the qualitative analysis software Atlas.Ti 8.0 and the final analysis was supervised for one of the research team (A.A.A.-S.). The text fragments were labeled in 201 codes and then grouped into three categories.

### 2.4. The Methods Integration Approach

Triangulation methods were applied, achieving the integration of both substudies [19]. A conceptual map was formulated, identifying the factors influencing the QOL on several levels (singular, academic, social, contextual), according to individuals’ opinions and considering the particularities of this specialists’ group in Colombia.

### 2.5. Ethics

This study was approved by the bioethics research committee of the Faculty of Dentistry of the University of Antioquia (Act 08/2020, Concept N° 59). Individuals’ participation was voluntary. Considering that remote means were used for the fieldwork (because of the isolation measures during the COVID-19 pandemic), participants gave their consent, which was explained on the first page of the survey (quantitative) and was read and approved orally during the recording of the interviews and FGs (qualitative). Confidentiality was guaranteed throughout the research process following Colombian regulations (Resolution No. 008430/1993—Ministry of Health and Social Protection).

## 3. Results

### 3.1. Quantitative Aspects

#### 3.1.1. Sociodemographic, Labor, Health Profile, and QOL of Participants

The sociodemographic characteristics of the population participating in the study are shown in Table 1. The mean age of respondents was 47 ± standard deviation (SD) 10 years. Most of them were women, more than half were either married or cohabitated, two-thirds were from high socioeconomic status; a little more than 70% live in their own homes, and most have a vehicle. Considering labor characteristics, the median (interquartile range (IQR)) of the experience as specialists was 16 (±IQR 19) years. More frequently, the respondents carried out teaching and healthcare activities, and almost three-quarters had written contracts with their employers. Most participants had independent contracts or worked on a percentage basis, with a median of 60% (±IQR 10%). Participants reported a mean of 37 ± SD 15 h/week and a median of 2 (±1 IQR) days off; 70% have an average monthly income of more than 4 million Colombian pesos (about 950 dollars or more). They work with a median of 3 (±4 IQR) workplaces and a good deal considers that the salary allows them to cover basic needs, cover unforeseen expenses, are well paid, and are satisfied with their work-related activities. Similarly, for a good part of the participating population, the specialist degree has allowed them to join the labor market and is consistent with the academic curricula.

Concerning the QOL variables, the median of the scores of the different dimensions of the WHOQOL-BREF surpassed 62 points, being higher in the physical and lower in the environmental dimensions. Considering health variables, more than 60% referred to the practice of sports, the majority reported stress at work, a fifth reported their general health as poor, and a little more than two-thirds reported poor mental health. Low social support was referred to in 13% of cases (Table 1).

#### 3.1.2. Sociodemographic, Labor, and Health Aspects That Are Related to the QOL in Different Dimensions

Table 2 shows the bivariate correlations between the QOL dimensions of the WHOQOL-BREF instrument and the quantitative variables in the study sample. Statistically significant and negative correlations were found between the physical dimension and the value of the percentage in labor contracts (the higher the percentage, the lower the quality of life and vice-versa). The same was observed for the psychological dimension and the number of working hours per week. A statistically significant and positive correlation was found between the psychological dimension and the number of rest days per week.

Table 3 shows the differences in QOL levels between the various sociodemographic, labor, and health variables. For the physical dimension, statistically significant differences (*p* < 0.05) were found in the scores according to the socioeconomic status (>middle), in terms of the opinion if the salary is well paid (>yes), considering the sports practice (>yes), and depending on the perception of their general and mental health (>good). Regarding the psychological dimension, statistically significant differences were observed (*p* < 0.05), according to the type of housing (>own housing), the practice of clinical activities (>no), the monthly income, the practice of sport (>yes), and depending on the perception of their general and mental health (>good). Concerning the social relationships dimension, statistically significant differences were observed according to the monthly income, considering the level of job satisfaction (>satisfied), the practice of sports (>yes), their general and mental health (>good), and the social support (>normal). Finally, considering the environmental dimension, statistically significant differences were found in terms of clinical activities (>no), monthly income, level of job satisfaction (>satisfied), the practice of sports (>yes), their mental health (>good) and the social support (>normal).

#### 3.1.3. Potential Explicative Factors for the Dimensions of the QOL

In the multivariate linear regression models for each domain of the WHOQOL-BREF instrument (Table 4), it was observed that the main factors reported as associated with QOL were general health as poor in the case of the physical and social relationships dimensions (it was negatively associated, which means that poor health is associated with a lower QOL score). Reporting poor mental health is negatively associated with QOL in the physical and psychological dimensions. Having an independent or service provision contract was negatively associated with the physical dimension. Having low social support was negatively associated with QOL in the social relationships dimension and being dissatisfied at work was negatively associated in the case of the environmental dimension.

### 3.2. The Participants’ Discourses in the Qualitative Sub-Study

The analysis process carried out for the interviews and FGs permitted the generation of three main categories, which are described in detail below:

#### 3.2.1. QOL and Health: Multiple Facets, Determinants, and Dimensions

The QOL from the discourses of the participating population exhibits numerous facets that denote a balance in the different spheres of human development and that lead to general well-being in physical, psychological, emotional, spiritual, and social aspects. It is a relative concept in which, the satisfiers, needs, and expectations are intermingled against scales of values and standards that allow the concept to be differentiated and that become their own to the extent that objectives are achieved and the people can enjoy elements of the daily life, reconciling time to be with the family and being able to enjoy other activities not related to their profession and work (Table 5, 1a, 1b, 1c).

In the perceptions of the participants, the appreciation of the QOL was good (Table 5, 1d). However, they recognized that some characteristics or factors could influence this QOL, highlighting those that contribute positively, such as having job benefits, having free time, practicing sports, and experiencing constant academic and professional development. As negative factors, they detached elements of their personal experience, as well as those related to the social and political context of the country, and the characteristics of the labor market that limited their performance as specialists (Table 5, 1e).

QOL cannot be separated from the concept of health. For the participants, self-care becomes important as a fundamental element that contributes to identifying their problems and solutions. The labor field can be an element that generates emotional loads and physical fatigue, which is related to the QOL (Table 5, 1f).

According to the experience of participants, the role played by social support networks is a factor that is related to the improvement of QOL. They highlighted the role of the family and the different academic and trade associations. However, in this last aspect, they recognized that there was low participation of specialists in this type of association when compared to others (Table 5, 1g, 1h).

During the fieldwork period, the country was experiencing a moment of mandatory social isolation due to the COVID-19 pandemic. In the voices of the participants, the relationship of this disease with the QOL is timidly mentioned, since when the qualitative component was carried out with the focus groups, the restrictions to work in dental clinics had already ended. However, they mention how some changes were presented at the professional level and in some cases, rethinking the career and work plans and perceived symptoms of anxiety and affectation in mental health (Table 5, 1i).

#### 3.2.2. Encounters and Disagreements between the Proposal of Postgraduate Curricular Training and the Labor and Social Field of the Specialists

The postgraduate training at the Faculty of Dentistry of the University of Antioquia has had some transformations in its curricular dynamics, from a specialization focused on care for children with an emphasis on maxillary orthopedics. This situation has generated some difficulties, mainly concerning access to the labor market in some public and private health institutions with the diploma offered by the university, because there is a lack of trust considering the professional skills of the specialist and the name of the postgraduate training (Table 5, 2a). Similarly, some difficulties could arise due to the recognition of titles by the Ministries of Education and Health when services are enabled in dental offices, but these problems are isolated and cannot be generalized (Table 5, 2b). Another aspect is related to the difficulties of integration and recognition with some scientific societies and guilds due to the denomination of the current diploma (Table 5, 2c).

The University of Antioquia enjoys good acceptance in the region and this can be an important factor for specialists to have professional recognition in the area of pediatric dentistry and maxillary orthopedics and some cases, facilitate interdisciplinary work (Table 5, 2d). The participants mentioned that the area of pediatric dentistry is more undervalued and that the economic remuneration is much lower than when working with maxillary orthopedics (Table 5, 2e). In this last aspect, they compete with some orthodontic specialties in educative institutions that also offer training in maxillary orthopedics. It is recommended to improve the identity of the postgraduate considering the professional skills, the trends in the labor market, and seeking greater visibility, leadership, and international projection (Table 5, 2f).

#### 3.2.3. An Itinerant Clinical Specialization

The experiences of the study population denote a profession whose employment and work conditions are related to the fact that a good part works in different jobs, many of them located in municipalities outside of Medellin and its metropolitan area. For this reason, they receive diverse incomes and have different types of contracts (Table 5, 3a). Some of these contracts have disadvantages, for instance, the mode of labor contract or percentage, which does not have labor benefits such as health affiliation, pensions, and occupational risks, which must be assumed entirely by the professional. In this sense, the benefits of a fixed contract are weighed against the short-term economic benefits of a labor contract (Table 5, 3b, 3c). However, they stated that when they have this type of contract, they have greater independence to negotiate work schedules and reconcile free time to carry out other activities, according to their personal, family, and social priorities (Table 5, 3d).

From the participants’ perspective, the working conditions of specialists in pediatric dentistry and related areas are much better when compared to those received as general dentists, but are inferior when compared to other clinical specialties. They perceived that they must attend to a greater volume of patients, with a greater workload to obtain an income that satisfies their own needs and contributes to improving their QOL (Table 5, 3e). In some cases, the study participants recognized that assisting the child population in different fields of knowledge requires a lot of concentration and this increases certain demands. This can be reflected in a higher workload (Table 5, 3f). Closely related to the specific conditions of the workplace, some signs and symptoms of musculoskeletal disorders are perceived, but it is not commented that they have sought attention by occupational medicine (Table 5, 3g).

### 3.3. The Integration Methods Approach

#### A Conceptual Map for Understanding the QOL in Participants

**Figure 2:**
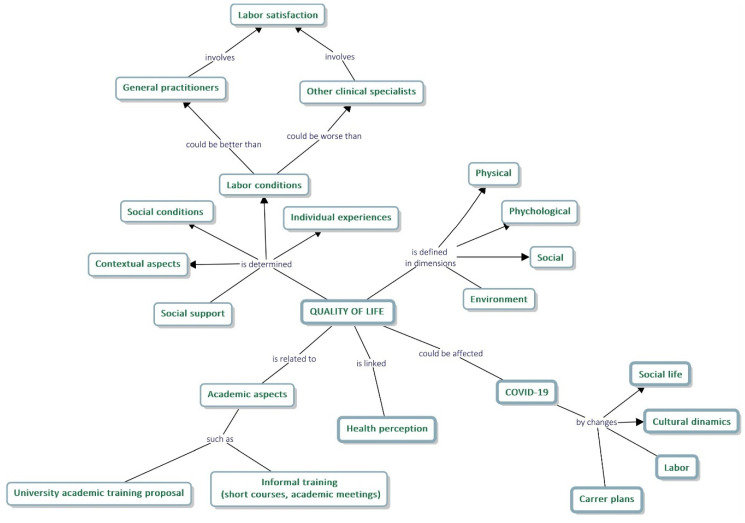
A conceptual explanatory map for understating the factors influencing QOL in the study population. Medellin, 2021–2022. Elaborated by using Cmaptools 6.03.01.

Figure 2 shows the conceptual map that integrates the main findings of both sub-studies. In short, QOL was defined by participants in different dimensions, with high scores (according to the WHOQOL-BREF). However, this QOL is determined by factors related to the social, economic, and labor contexts of clinical specialties such as pediatric dentistry, both internally and in comparison, with other specialties. Similarly, and in line with the qualitative results, aspects of academic training in the specialty are mentioned, and more timidly the association of the COVID-19 pandemic with some social, labor, and cultural dynamics, although the assessment of this relation was not the aim of the study.

## 4. Discussion

### 4.1. Main Findings

This study analyzed individual, social, cultural, academic, and work factors that influence the QOL of a particular group of pediatric dentistry clinical specialists and those who graduated from a public university of academic leadership in the country. Regarding the profile of the study participants, they are graduated from the program that the University of Antioquia has offered in the area of pediatric dentistry for several decades, presenting over time, an evolutionary process that involves changes in the denomination, the curricula, and the academic and occupational profiles. The participants in this study have completed different versions of the specialization program. Although these participants had different profiles, conditioned by the version of the curricula in force in each cohort, there were no contradictory testimonies during the development of the surveys, the SIs, and FGs regarding the perception of QOL.

### 4.2. Possible Explanations for the Findings from an International Perspective

One of the elements that are interesting at first glance is the female participation in the study (81%). This participation is much higher when compared to a similar study conducted on orthodontists at the same university [16]. During the last decades, there have been feminization processes in many professions, and dentistry is no exception [26]. This supports the need for analyzing social and health indicators from a gender perspective [27], which constitutes an analytical category that makes it possible to highlight situations of inequality and equity between men and women. In this particular study, taking into account the size of the sample, general analyses were made adjusting for the sex variables. On the other hand, although in the qualitative sub-study the possible differences between men and women were inquired into, the discourses of the participants did not mention it in depth as an element of greater importance compared to other categories and determinants. In any case, it is possible that in other groups of professionals and considering other phenomena to be investigated, these types of differences were observed.

The median of the scores in the different dimensions of the WHOQOL-BREF surpassed 62 points, being higher in the physical dimension and lower in the environmental dimension. This suggests a good QOL in the study population. Studies carried out in other social and geographical contexts that use the same instrument showed some differences when each of its dimensions is analyzed.

A study in Brazil conducted on public sector dentists found that the social domain had the highest scores and the environmental domain had the lowest scores (taking into account the median scores, with scores between 60 and 75) [7]. One of the most recent studies carried out in Colombia on orthodontists showed a higher score in the psychological dimension and a lower score in the physical dimension (with scores between 57 and 71) [16]. A study carried out on dentists who teach in Indian hospitals showed that the social relationships dimension obtained the highest scores, and that the dimension related to the environment obtained the lowest. It is important to mention they used the mean as a measure of central tendency and the scores surpass 66 points, indicating a good overall QOL [8]. In the United Arab Emirates, a study conducted on general dentists and specialists in private practice showed that the social domain obtained the highest scores and the physical domain had the lowest scores. In a similar way to India, the scores surpassed 69 points, indicating a good overall QOL [9]. The findings of these studies showed that the perception of QOL is conditioned by the particular aspects of the country and the sample considered in each study.

Complementing the above, it is estimated that the lower score reported in the population participating in our study concerning the environmental domain and as found in the studies carried out in Brazil [7] and India [8] could be due to factors generators of inequality and that is related to financial resources, physical security, health, and recreation/leisure opportunities of each population (as presented in the questions that assess this domain in the WHOQOL-BREF instrument). In this sense, the qualitative findings highlighted the influence of social support networks (colleagues and friends) and family on QOL and the importance of having spaces to share with others, and, on the other hand, practicing sports was a variable of importance that increases QOL according to the quantitative results, as well as social support. A study carried out in Sweden shows that dentists who show greater professional control and social support have less workload (this can be considered as a direct relationship with QOL) [28]. In the same way, performing sports or physical activity and enjoying leisure and free time spaces influence the state of physical and mental health, as shown by studies carried out on the research topic [29,30].

The multivariate analysis by linear regression showed that labor and percentage contracts for the provision are negatively associated with QOL, as the job unsatisfaction. Similarly, the bivariate analyses showed associations for QOL between the level of monthly income and the fact of considering whether the job was well paid. When this information is cross checked with the qualitative findings, the participants perceived that specialization is an itinerant profession, with fewer labor benefits compared to permanent contracts, but that these working conditions are compensated by the fact that they optimize the time to reconcile their work and personal life.

Several elements of analysis must be taken into account in these findings. First of all, dentistry is a liberal profession and it is common for specialists to work much more in private practice than in public service [6]. Next, the general conditions of specialists could be considered much better when compared to general dentists, and in this regard, a focused systematic review show that specialists have greater job satisfaction and among them, pediatric dentists [31], and one study in the United States reflected the same results [32]. It should be noted that 89% of the study participants in Medellin were satisfied with their employment situation. They recognized in the discourses the possibility that their general conditions could be different from other specialties such as orthodontics, although with comparable scores according to the WHOQOL-BREF, as observed in one study carried out in the same city [16]. Finally, a process of personal and labor adaptation is observed in the specialists who work in the private sector, and in those who work in activities related to teaching, according to their interests, motivations, and expectations.

The relationship between health status and QOL was observed in the study findings, wherein in the bivariate analyses and the multivariate models, the lowest QOL scores were associated with a perception of poor physical and mental health (it is important to mention that one-third of respondents to the survey reported poor mental health, and one-fifth also reported self-perceived poor general health). In this association, multiple factors and determinants exist and operate at different levels. In addition, the discourses of the participants complemented this relation where the workload generated by the dental care of pediatric patients and their families can be a generator of stress and emotional loads. Quantitative data provided in the cross-sectional survey showed that 14.5% of respondents reported stress at work.

The association between the health status (by using different general and mental indicators) and the QOL also was observed in a Brazilian study, where a relationship between the domains of the used instrument for evaluating QOL and the current health status [7]. A systematic review focused on the main physical and mental complaints of the dentists indicated the influence of individual, social and workplace-related factors that act as stressors [11]. A study in Sweden complements these findings, where there is a relationship between the health status and the physical and psychological demands that are generated by the dental profession [28]. A multicenter study conducted in 21 countries shows other indicators that may be indirectly related to QOL, such as happiness, life satisfaction, and psychological well-being [33]. Their main findings provided academic support about the relationship between physical and mental stressors with QOL and secondly, they conclude that there are factors related to the country of origin and other social aspects involved in the perception of their feelings and well-being. Further research could broaden specific knowledge about risk factors in the workplace, using questionnaires focused on occupational health.

### 4.3. Strengths, Weaknesses and Scope of This Study

In the interpretation of the results, the periods of data collection, the study objectives, and the questions included in the fieldwork instruments should be considered. This is of interest as there are explanations for the findings that were not necessarily related to the framework of the COVID-19 pandemic in these dental healthcare workers. The qualitative findings scantily mentioned the relation of the pandemic on the dynamics of the participating population. In this regard, the scientific literature has drawn attention to the influence of the pandemic on the modification of dental practice and career plans [34,35], on labor well-being [36], and physical and mental health indicators [37]. Further studies may investigate the conditions of general dentists and specialists, in this new world order after almost two years of the start of the pandemic, and where oral health professionals have experienced processes of adaptation to this situation.

The social context of the specialization has evidenced numerous curricular and academic changes that have influenced the dental practice of these professionals, and that has had an influence not only in the form of denomination and study plan but also on professional and labor competencies, which have not been well interpreted in Colombia and that require further discussion and analysis. However, a little more than 80% of the participants were satisfied with the professional diploma and with the training received to face the labor market although this was not a factor that influenced the QOL, neither in the multivariate models nor in the discourses of the participants, since there were found protective factors such as the acceptance of the university in the region, although they recognized that the profession is less valued concerning others. In this sense, the importance of changing paradigms in the educative models for this specialization is well acknowledged [38], and conducting comparative analyses of the training aspects of these professionals in educative institutions of Latin America, has an integral approach and review of the relation with QOL.

The above findings highlight the importance of discussing the main limitations and strengths of this study with a view to its interpretation. Although to the best of our knowledge, it is the first study in Colombia focused on this population, the results cannot be applied from the general population of specialists in the area of pediatric dentistry and related fields in Colombia, since it is not a population-based study with probabilistic/representative sampling. The analyses conducted about the effect size employing the Eta squared showed values between 0.001 and 0.279 (the sociodemographic and labor variables tended to have a small effect size and the health variables had a medium effect size). This situation agrees with the multivariate analyzes where variables with greater predictive capacity were found (especially those related to health indicators), and with the discourses in the FGs. Possible explanations lie in factors related to the size and type of sample (relative homogeneity in demographic and labor aspects). The nature of the study does not allow causal relationships to be established, but rather associations between variables and holistic understandings between categories of analysis. Regarding the response rate of the participants, 62 participants responded from a database of 108 specialists who graduated from the institution. This may affect the study findings since the decision to answer the survey may be related to the perceptions of the QOL and the results could be underestimated. In this regard, a literature review showed that survey response rates among healthcare workers tend to be lower than those of the general population due to their demanding work schedules [39]. As strengths, recognizing that the data collection instruments and the mixed nature of the research were carefully planned and have internal validity processes, both by pilot test and by the consistency and validation of the QOL questionnaires, as well as with reliability and rigor in the qualitative techniques used.

Accepting the limitations of this research, and taking into account the previous scientific literature on related topics, the findings constitute an important element to understanding the social reality of clinical specialists in the area studied in the country and are a gateway for other studies of greater scope and depth. In the same way, it seems important to establish strategies to monitor the situation of graduates in educational institutions that train human talent in oral health.

## 5. Conclusions

The QOL of the study participants is categorized as good, considering the instrument scores and the information shared. However, given its multifactorial and multidimensional nature, it can be affected in its physical, mental, social, and environmental dimensions by individual, social and contextual factors that operate at different levels, and that are differential among the same specialists. Sociodemographic, work, and health conditions affected QOL to a greater extent, especially having low social support, independent or labor contracts, and perceiving poor physical and mental health.

## Figures and Tables

**Table 1:** Sociodemographic, labor, quality of life and health characteristics of the study population. Medellin, 2021–2022 (*n* = 62).

Variables	*n*	%
Sociodemographics		
Sex		
Females	50	80.6
Males	12	19.4
Age ^a^		
Mean (±SD)	47.0	10.4
Marital Status		
Single	18	29.0
Married-Cohabitated	37	59.7
Separated	7	11.3
Socioeconomic status		
Middle	20	32.3
High	42	67.7
Housing		
Own	44	71.0
Rented	12	19.4
Other	6	9.7
Vehicle		
Yes	55	88.7
No	7	11.3
Number of people in charge ^a^		
Median (IQR)	1.0	2.0
Labor conditions		
Years of experience as a dentist ^a^		
Median (IQR)	22.5	16.0
Years of experience as a specialist ^a^		
Median (IQR)	16.0	19.0
Labor activity ^b^		
Teaching/Research	53	85.5
Clinical assistance	34	54.8
Administrative	7	11.3
Other	3	4.8
Written contract (*n* = 61)		
Yes	45	73.8
No	16	22.2
Type of Contract ^b^		
Permanent	17	27.4
Temporal	9	14.5
Independent	32	51.6
Provision of services	22	35.5
Percentage rent	40	64.5
Percentage rent value ^a^		
Median (IQR)	60.0	10.0
Working hours per week ^a^		
Mean (±SD)	37.3	14.6
Resting days per week ^a^		
Median (IQR)	2.0	1.0
Monthly income (Colombian peso) ^c^		
<3,000,000 (U$792)	6	9.7
3,000,001–4,000,000 (U$793–U$1056)	12	19.4
4,000,001–5,000,000 (U$1057–U$1319)	12	19.4
5,000,001–6,000,000 (U$1320–U$1582)	10	16.1
>6,000,001 (U$1583)	22	35.5
Number of workplaces ^a^		
Median (IQR)	3.5	4.0
Does your current salary allow you to cover your basic needs, and those of the people who depend on you?		
Yes	58	93.5
No	4	6.5
Does your current salary allow you to cover unforeseen important expenses?		
Yes	54	87.1
No	8	12.9
Do you think your salary is well-paid for the work you do and the time you dedicate to it?		
Yes	41	66.1
No	21	33.9
Annual frequency of participation in events of training and unformal education ^a^		
Median (IQR)	3.5	4.0
Do you consider that the degree you obtained during your training period as a specialist is consistent with the curricula?		
Yes	52	83.9
No	10	16.1
Do you consider that the specialist degree you obtained has allowed you to be adequately linked to the labor market?		
Yes	54	87.1
No	8	12.9
Labor Satisfaction		
Satisfied	55	88.7
Unsatisfied	7	11.3
Quality of Life (QOL)		
Physical ^a^		
Median (IQR)	71.4	25.0
Psychological ^a^		
Median (IQR)	68.8	16.7
Social relationships ^a^		
Median (IQR)	66.7	33.3
Environment ^a^		
Median (IQR)	62.5	16.4
Health		
Sport Practice		
Yes	39	62.9
No	23	37.1
Stress in the workplace		
Yes	53	85.5
No	9	14.5
Self-perceived health		
Good	49	79.0
Poor	13	21.0
Social support (Duke-UNC-11)		
Normal	54	87.1
Low	8	12.9
Mental Health (GHQ-12)		
Good	39	62.9
Poor	23	37.1

^a^ Kolmogorov-Smirnov test for Normality. Skewness and kurtosis values for the domains of the WHOQOL-BREF domains are: physical (−0.046; −1.047); psychological (−0.121; −0.348); social relationships (0.138; −0.726), environment (0.278; −0.372). For the other study variables, the values are: age (0.314; −0.864); number of people in charge (0.509; −0.413); years of experience as a dentist (0.154; −1.095); years of experience as a specialist (4.441; 27.961); percentage rent value (−0.421; 0.138); working hours per week (−0.278; −0.065); resting days per week (0.449; −0.798); number of workplaces (1.685; 3.468). Variables with normal distribution: age (years) and working hours per week. ^b^ Non-mutually exclusive percentages. ^c^ Dollar values between parenthesis (at the time of the fieldwork). IQR: interquartile range.

**Table 2:** Bivariate correlations between the WHOQOL-BREF dimensions of quality of life and the sociodemographic, labor and health variables in the study sample. Medellin, 2021–2022 (*n* = 62).

Variables	WHOQOL-BREF Dimensions (QOL)			
	Physical	Psychological	Social Relationships	Environment
Age	−0.072	0.044	−0.048	0.133
Number of people in charge	−0.218	−0.026	−0.118	−0.022
Years of experience as a dentist	−0.005	0.085	−0.024	0.147
Years of experience as a specialist	−0.041	0.033	−0.071	0.067
Percentage rent value	−0.338 *	−0.220	−0.058	−0.118
Working hours per week	−0.198	−0.272 *	−0.184	−0.096
Resting days per week	0.061	0.260 *	0.001	0.113
Number of workplaces	−0.029	−0.070	0.034	−0.127
Annual frequency of participation in events of training and unformal education	−0.029	−0.070	0.034	−0.127

Spearman’s rank correlation coefficient. * *p*-value < 0.05.

**Table 3:** Differences in QOL levels according to the various sociodemographic, labor, and health variables in the study sample. Medellin, 2021–2022 (*n* = 62).

Variables	WHOQOL-BREF Dimensions (QOL)															
	Physical				Psychological				Social Relationships				Environment			
	Me	IQR	*p*-Value	Eta Squared	Me	IQR	*p*-Value	Eta Squared	Me	IQR	*p*-Value	Eta Squared	Me	IQR	*p*-Value	Eta Squared
Sociodemographics																
Sex																
Females	67.9	22.3	0.469	0.007	70.8	20.8	0.713	0.007	66.7	33.3	0.921	0.001	62.5	16.4	0.893	0.005
Males	80.4	33.0			66.7	19.8			62.5	25.0			60.9	20.3		
Marital Status																
Single	73.2	25.9	0.858	0.004	68.8	27.1	0.766	0.008	70.8	29.2	0.754	0.008	62.5	17.2	0.737	0.015
Married-Cohabitated	71.4	23.2			66.7	14.6			58.3	37.5			62.5	14.1		
Separated	64.3	28.6			70.8	16.7			75.0	33.3			62.5	28.1		
Socioeconomic status																
Middle	80.4	20.5	0.029 *	0.073	72.9	30.2	0.214	0.01	75.0	33.3	0.404	0.007	60.9	18.0	0.785	0.002
High	64.3	18.8			66.7	12.5			58.3	27.1			62.5	16.4		
Housing																
Own	71.4	21.4	0.168	0.064	70.8	16.7	0.023 *	0.129	66.7	33.3	0.132	0.062	62.5	18.8	0.090	0.078
Rented	69.6	26.8			66.7	17.7			58.3	22.9			64.1	14.8		
Other	58.3	23.2			54.2	19.8			50.0	14.6			50.0	16.4		
Vehicle																
Yes	71.4	25.0	0.244	0.024	66.7	16.7	0.695	0.001	66.7	33.3	0.983	0.001	62.5	15.6	0.828	0.003
No	82.1	35.7			75.00	37.5			58.3	58.3			65.6	28.1		
Labor conditions																
Labor activity																
Teaching/Research																
No	64.3	24.1	0.060	0.055	66.7	21.9	0.292	0.008	58.3	39.6	0.122	0.038	57.8	11.7	0.039 *	0.053
Yes	76.8	25.0			70.8	16.7			66.7	27.1			64.1	16.4		
Clinical assistance																
No	75.0	35.7	0.362	0.016	79.2	14.6	0.027 *	0.074	66.7	20.8	0.450	0.006	68.8	15.6	0.048 *	0.069
Yes	67.9	25.0			66.7	14.6			58.3	33.3			59.4	14.1		
Administrative																
No	71.4	25.0	0.862	0.001	70.8	20.8	0.679	0.001	66.7	33.3	0.382	0.011	62.5	15.6	0.303	0.022
Yes	60.7	28.6			66.7	8.3			75.0	25.0			65.6	28.1		
Other																
No	71.4	25.0	0.664	0.006	66.7	16.7	0.394	0.020	66.7	33.3	0.450	0.011	62.5	15.6	0.265	0.045
Yes	71.4	--			79.2	--			66.7	--			75.0	--		
Written Contract																
Yes	75.0	21.4	0.095	0.054	70.8	14.6	0.564	0.009	66.7	33.3	0.286	0.022	62.5	14.1	0.651	0.001
No	58.9	34.8			68.8	20.8			62.5	33.3			60.9	23.4		
Type of Contract																
Permanent																
No	71.4	26.8	0.393	0.011	70.8	16.7	0.067	0.049	66.7	37.5	0.639	0.004	62.5	18.8	0.994	0.001
Yes	64.3	19.6			66.7	14.6			58.3	25			59.4	14.1		
Independent																
No	73.1	25	0.15	0.031	70.8	16.7	0.257	0.021	70.8	37.5	0.065	0.064	64.1	14.8	0.088	0.001
Yes	64.3	21.4			66.7	19.8			58.3	25.0			59.4	15.6		
Provision of services																
No	73.2	24.1	0.479	0.009	70.8	12.5	0.871	0.002	66.7	31.3	0.853	0.001	60.9	18	0.976	0.001
Yes	62.5	26.8			66.7	30.2			62.5	37.5			62.5	17.2		
Percentage rent																
No	66.1	25.9	0.497	0.009	70.8	16.7	0.625	0.003	62.5	25	0.801	0.001	59.4	21.9	0.773	0.005
Yes	71.4	21.1			66.7	19.8			66.7	33.3			62.5	15.6		
Temporal																
No	67.9	25	0.207	0.022	66.7	16.7	0.191	0.015	58.3	33.3	0.251	0.018	62.5	15.6	0.244	0.015
Yes	78.6	14.3			75	12.5			66.7	29.2			62.5	14.1		
Monthly income (Colombian peso)																
<3,000,000 (U$792)	60.7	9.8	0.055	0.151	50.0	13.5	0.017 *	0.232	50.0	25.0	0.050	0.156	50	17.2	0.016 *	0.216
3,000,001–4,000,000 (U$793–U$1056)	78.6	29.5			75.0	24.0			83.3	39.6			71.9	14.8		
4,000,001–5,000,000 (U$1057–U$1319)	69.6	31.3			66.7	26.0			62.5	25.0			59.4	14.1		
5,000,001–6,000,000 (U$1320–U$1582)	80.4	24.1			70.8	16.7			70.8	22.9			60.9	19.5		
>6,000,001 (U$1583)	66.1	28.6			66.7	9.4			58.3	33.3			62.5	16.4		
Does your current salary allow you to cover your basic needs, and those of the people who depend on you?																
Yes	71.4	25.0	0.751	0.003	68.8	16.7	0.814	0.001	66.7	33.3	0.923	0.001	62.5	18.8	0.351	0.015
No	71.4	28.6			68.8	16.7			58.3	25.0			56.3	18.0		
Does your current salary allow you to cover unforeseen important expenses?																
Yes	71.4	25.9	0.138	0.032	70.8	16.7	0.151	0.031	66.7	33.3	0.841	0.001	62.5	18.8	0.513	0.007
No	60.7	16.1			64.6	15.6			58.3	33.3			59.4	10.9		
Do you think your salary is well-paid for the work you do and the time you dedicate to it?																
Yes	75.0	25.0	0.019 *	0.092	70.8	16.7	0.127	0.037	66.7	33.3	0.297	0.023	62.5	20.3	0.078	0.057
No	60.7	17.9			66.7	25.0			58.3	33.3			56.3	14.1		
Do you consider that the degree you obtained during your training period as a specialist is consistent with the curricula?																
Yes	71.4	25.0	0.848	0.001	70.8	16.7	0.525	0.003	66.7	33.3	0.344	0.014	62.5	18.8	0.351	0.015
No	71.4	25.9			64.6	16.7			58.3	37.5			57.8	13.3		
Do you consider that the specialist degree you obtained has allowed you to be adequately linked to the labor market?																
Yes	71.4	25.0	0.809	0.001	66.7	13.5	0.225	0.023	58.3	25.0	0.208	0.025	62.5	16.4	0.941	0.001
No	71.4	30.4			77.1	30.2			79.2	41.7			60.9	22.7		
Labor Satisfaction																
Satisfied	71.4	21.4	0.253	0.018	70.8	16.7	0.058	0.060	66.7	33.3	0.039 *	0.061	62.5	15.6	0.004 **	0.109
Unsatisfied	57.1	10.7			54.1	16.7			50.0	16.7			53.1	6.3		
Health																
Sport Practice																
Yes	78.6	21.4	<0.001 ***	0.246	70.8	16.7	0.020 *	0.074	75.0	33.3	0.062	0.045	59.4	12.5	0.012 *	0.099
No	60.7	17.9			62.5	16.7			58.3	25.0			62.6	21.9		
Stress in the workplace																
Yes	71.4	25.0	0.984	0.001	70.8	14.6	0.469	0.019 *	66.7	41.7	0.694	0.004	62.5	17.2	0.547	0.001
No	60.7	41.1			66.7	29.2			66.7	29.2			59.4	20.3		
Self-perceived health																
Good	75.0	23.2	<0.001 ***	0.218	70.8	12.5	<0.001 ***	0.279	66.7	25.0	<0.001 ***	0.234	62.5	21.9	0.133	0.048
Poor	57.1	12.5			54.2	14.6			41.7	8.3			59.4	12.5		
Social support (Duke-UNC-11)																
Normal	73.2	21.4	0.051	0.061	70.8	16.7	0.063	0.072	66.7	33.3	0.003 **	0.121	62.5	16.4	0.002 **	0.152
Low	57.1	18.8			60.4	26.0			45.8	14.6			48.4	6.3		
Mental Health (GHQ-12)																
Good	78.6	21.4	0.001 **	0.187	70.8	12.5	0.003 **	0.162	75.0	33.3	0.004 **	0.130	62.5	18.8	0.012 *	0.111
Poor	60.7	10.7			62.5	20.8			58.3	25.0			56.3	18.8		

Mann-Whitney U test for dichotomous variables, Kruskal-Wallis test for polychotomous variables. IQR: interquartile range. * *p*-value < 0.05; ** *p*-value < 0.01; *** *p*-value <0.001.

**Table 4:** Lineal regression models for the scores of the WHOQOL-BREF dimensions of quality of life according to the variables included in the study sample. Medellín, 2021–2022 (*n* = 62).

WHOQOL-BREF Dimensions (QOL)	Variables Included in the Lineal Regression Model	Determination Coefficient % (R2%)		Change of R2%	*p*-Value Change of R2%	Non-Standardized Regression Coefficient	Standardized Regression Coefficient	*p*-Value	F-Value	*p*-Value (Model)	Durbin-Watson Statistic
		Unadjusted	Adjusted								
Physical	Independent contract	55.0	49.0	6.0	0.04 *	−8.43	−0.30	0.015 *	10.470	<0.001 ***	1.832
	Provision of services contract					−7.01	−0.42	0.040 *			
	Self-perceived health (poor)					−12.23	−0.25	0.009 **			
	Mental Health (poor)					−8.48	−0.28	0.040 *			
Psychological	Mental Health (poor)	23.0	21.0	22.0	0.002 **	−13.57	−0.48	0.002 **	11.087	0.002 **	2.359
Social relationships	Self-perceived health (poor)	40.0	36.0	12.3	0.009 **	−27.74	−0.54	<0.001 ***	12.173	<0.001 ***	1.556
	Social support (low)					−23.73	−0.35	<0.001 ***			
Environment	Labor Satisfaction (Unsatisfied)	17.0	15.0	16.0	0.009 **	−11.86	−0.41	0.009 **	7.695	0.009 ***	2.342

* *p*-value < 0.05; ** *p*-value < 0.01; *** *p*-value < 0.001. Method for the multivariate lineal regression: Stepwise.

**Table 5:** Verbatim extracts from participants’ discourses: Focus groups (*n* = 3) and semi-structured interviews (*n* = 4).

Categories	Key Words	Verbatim Extracts form Participants’ Discourses
(1) Quality of life and health: multiple aspects, determinants and dimensions	Quality of life as balance	(a) “In my opinion quality of life means balance between different things. Not only what the other participant said about also having free time to spend in other things besides work, but also having balance in everything that represents the income one can earn in life, what one can do for others, or the interpersonal relationships one has with others. So, it means to have balance in all the different aspects: not only timewise, but also in the work and family aspects, as well as the different relationships that one can have with other people and with one-self. Then it’s the balance that you can bring about.“(Focus Group 03)
	Quality of life as well-being	(b) “Basically, it is perhaps that individual perception of well-being in all its comprehensive aspects, its social aspect, its family aspect, its economic and professional aspect. And let’s say that provided that the survival, the basic needs for living are met, from there everything adds up to that individual perception of quality of life.” (Focus Group 02)
	Quality of life and enjoyment of everyday things	(c) “Let’s say you can dedicate quality time to your family, to your home, to your priorities that can be academic, they can be personal, they can be recreational, but it is like allowing yourself that balance and allowing yourself to participate in different areas of life.” (Interview 01)
	Assessment of the quality of life.	(d) “Well, as an outsider I think they have a good quality of life. I see them all working in what they studied, busy, I see them actively participating in conferences, I see that they have their personal lives, their professional and family lives. I assume that they have a good quality of life, because as far as I know I have not met anyone who has had great difficulties, I mean as graduates in the development of their profession.” (Interview 04)
	Quality of life and basic needs met	(e) “Exactly, what I said before, starting from the fact that basic needs are met, which obviously includes health, housing, the financial part, from then on everything adds up to the well-being. But I think that the basic thing for me is like knowing that I have a roof, a meal, I am healthy, I mean, that my fundamental rights are fulfilled. From there on, if I get to have more time for myself, if I have more space for my personal accomplishment, I don’t know, that adds up to my well-being. But for me the basic thing is what we were talking about, the basic needs because they are fundamental for the quality of life.” (Focus Group 2)
	Quality of life and health	(f) “To complement what the doctor says, it comes first of all from me, from my person, from what I perceive to be the quality of life, but what determines it, I would say that my health is something important, because let’s say I have a medical condition that I have not been able to control, then surely the quality of life that I have will be different than if I were healthy, if medically I felt good and I had... If I were physically fit to do what I could do. I may want to go travelling, but I don’t know, I have a respiratory condition, so surely that will prevent me from being able to do some things I want to do, or even from working.” (Focus Group 01)
	Social support networks	(g) “And the other aspect is the whole academic part and everything that one manages to do, as a working group, that improves one’s quality of life, going to the university and meeting the people we meet and sharing how we share, I think it is important. That is fundamental for the quality of life, the academic part is a very important one, the compensation part is very important obviously, I consider myself lucky to have them all there as coworkers, because I believe that we have done some work groups where we get along all the time. That is very good for the quality of life. Truth be told, one goes to the university willingly and wanting to meet people and share with people, I think the quality of life is good, for us it is good, it seems to me.” (Focus Group 01)
	Under associativism and union participation	(h) “Historically our participation in that has been bad, if not terrible in the sense of... bad in the sense that we aren’t very willing to participate from the union point of view. How many presidents of the ACOP (Colombian Association of Pediatric Dentistry) have been from Universidad de Antioquia?”
	Quality of life and COVID-19	(i) “And in the professional part, it entails many changes because let’s say that not being able to do every day the work activities we were used to do notably alters the rhythm of life to which we were used, so it is like it takes even more capacity for adaptation in all senses.” (Interview 01)
(2) Agreements and disagreements between the postgraduate curriculum training proposal and the specialist work and social field	Confusion in the work field	(a) “(...) that it is not so easy for them to enter the workforce when they do so in a large institution where they function around the classic names of the specialties, and I think that we saw it when we started graduating these girls who graduated with only a degree in orthopedics. When you work in a private practice it is a little easier because you don’t have to, let’s say, prove to —I don’t know whether to say central or human resources—, prove that you have the capacity or that you can count on it... (meaning the degree of the specialty and the action field in dentistry) that you have the training to also do the management part (management of the pediatric patient).” (Focus Group 01)
	Recognition of the degree in the Ministry of Health and Education	(b) “So, I say that it is very difficult to agree on the degree name, whether it will be pediatric dentistry and orthopedics, orthopedics and pediatric dentistry, pediatric dentistry only, orthopedics only. But the only thing you could do would be to look for the names that are recognized, that when you go where the other participant was saying, the REPS (Registry of Health Service Providers), you find it, because if you don’t...”
	Approval in associations	(c) “They do not approve us, because our degree does not say Pediatric Dentistry, so for example we have gone twice in the last few years. I made an arrangement with XXX (president of an academic association) so he will see us, I told them about when we were dentistry and maxillary orthopedics, I don’t remember anymore. And we even had to deliver some letters to be accepted and we almost had to show, well, the curriculum and say yes, we do dentistry and research in dentistry pediatrics, all that.” (Interview 04)
	Institutional recognition	(d) “They approve us because there is a very important support from the Faculty of Dentistry, the Faculty of Dentistry has a recognition and a support from which we, so to speak, benefit.” (Interview 01).
	Low compensation of pediatric dentistry	(e) “(…) There is an area of pediatric dentistry that is not well paid, right? And when a person is in the process of choosing the specialization, I think a very important aspect is the economic compensation.” (Interview 01)
	Improving postgraduate visibility	(f) “Yes, but I also think that we do our best, that is, the program seems ideal to me as it is set out, it has many good things, but also I would think that we must also strengthen it from the point of view of bringing external teachers, which we are doing now, because we are very, very closed and we realized that only by bringing people who are not like ourselves, we can enrich ourselves, because if you do not freshen the postgraduate program, sometimes then you think that what you are doing is very good and you do not realize that there are better things.” (Interview 03).
(3) An itinerant specialization	Multiple jobs, different economic income	(a) “There is also that limitation that XXXX (referring to a colleague) mentions, that we have to work a lot, but it is also the limitation of the market fluctuation, we have a market that is very fluctuating. Therefore, we cannot have something fixed, do you know what I mean? I mean we cannot have a fixed income, not so much, because sometimes the market,—well, for people who work in several practices—varies every month, then is also that, the monthly income.” (Focus Group 03)
	Balance between fixed contract and other types of temporary contracts	(b) “In my case, and I think I speak for everyone, I have never worked under a fixed-term, or indefinite type of contract, that is, I haven’t had the social benefits paid to me. So, you put that in the scale over time, like saying, I can better distribute my time when I do not have that type of contract. So, let’s say it all evens out in the long run, I prefer the model I have, I work as a service provider in different places...” (Focus Group 02)
		(c) “For example since I joined the university, even part time, I know that I get this amount of money, then I know that I can get into debt, I know that I can pay for house payments, I don’t know if it is because of that part of economic stability of being a teacher or because in my case what is stable is being a teacher, then I link those things like, well, I know how much I earn in the month at least in the university and that allows me to play. The rest are more external things because the salary is very variable, but as I do know how much I earn, I think that economic stability allows me to have more peace of mind and also what I said, quality of life is to have material things, from the point of view of meeting my expectations and needs and I think that having a fixed-term contract allows me to do so.” (Focus Group 01)
	Social advantages of temporary contracts	(d) “It seems to me that both of them obviously have pros and cons, for example, that depends a lot on the way people see life. For example, I like to travel a lot and so it’s more convenient for me to be a service provider, but for example, there are also people who need more financial stability, and then a service-provider type of contract may not really allow them to have that stability in their life, I mean, that depends a lot, depending on the perspective that a person has about life, about their priorities.” (Focus Group 03)
	Economic compensation: comparison with other specialties	(e) “What I perceive is that I feel like we have to work more to perhaps have an economic compensation that if one were to compare with other specialties that right now in the market are more striking—I would say that it is that social part that frames aesthetics a lot, orthodontics—then I feel like you have to work more to have that compensation.” (Focus Group 01)
	Emotional burden	(f) “(…) And also on an emotional level, yes, for me, in my opinion, the difficult thing is not only the management in itself, the stress that a child causes in the management, but it’s more difficult to meet the expectations of parents, then that does create an emotional burden, stress. But, well, I don’t know, everything balances out, there is time then to dedicate a little to ease that load, to lower the work time, you have to try to balance things out so as not to get sick. But yes, I think there is a health burden at work.” (Focus Group 02)
	Occupational hazards and health	(g) “In general, I have had health problems associated with the posture I have when working, I don’t know if maybe it’s for working with children, I think that today I have a less adequate posture, less ergonomic and the spine suffers more and I think that in general dentistry is a risk factor, because whenever I went to an orthopedist, they didn’t even do tests, instead they told me: you are a dentist, that is why your back hurts”. (Focus Group 01)

## Data Availability

Quantitative data presented in this study are available upon reasonable request from the corresponding author.
